# How can physical activity promotion be optimised in general practice: a narrative review of the literature

**DOI:** 10.1007/s11845-025-03932-5

**Published:** 2025-03-21

**Authors:** Stephen Dolan, Andrew O’Regan

**Affiliations:** 1 Department of Education and Health Sciences, University of Limerick School of Medicine, Limerick City, Ireland; 2 Health Research Institute, University of Limerick, Limerick, Ireland

**Keywords:** Exercise is medicine, General practice, Physical activity, Primary care, Review

## Abstract

**Background:**

Physical inactivity is a significant contributor to preventable chronic health conditions worldwide. General practice has been identified as a setting to improve physical activity levels through exercise promotion during consultations. However, physical activity promotion in general practice is unstructured and suboptimal.

**Aims:**

The aim of this study is to review the literature pertaining to factors that influence physical activity promotion in general practice and to answer the research question: what are the experiences of patients and GPs with physical activity promotion in consultations?

**Methods:**

Online databases were searched for relevant papers using predetermined inclusion and exclusion criteria. Papers retrieved were original research only, involving patients, general practitioners or practice nurses. A PRISMA approach to study selection was followed.

**Results:**

Of 464 papers retrieved, 20 were included in the review. Physical activity promotion is acceptable to patients but some do not appreciate its health benefits. A personalised approach is important to patients, including tailored advice and setting meaningful goals. Studies involving general practitioners and practice nurses report that they are aware of the importance of physical activity and their role in its promotion, but time is the primary barrier. Strategies identified include demedicalisation of physical activity, resources such as patient handouts as well as signposting to community initiatives, social prescribers and collaboration with exercise professionals.

**Conclusion:**

General practice has the potential to effectively promote physical activity but system- and practitioner-level changes are necessary to achieve meaningful change.

## Introduction

Globally, 7.2% of deaths are attributable to physical inactivity [1], while physically active older adults are at a reduced risk of all-cause and cardiovascular mortality, as well as chronic health conditions, including ischaemic heart disease, diabetes mellitus, frailty and certain types of cancer [2]. Currently, one-third of adults worldwide do not achieve the minimum levels of aerobic physical activity (PA) for health, recommended by the 2020 WHO Physical Activity and Sedentary Behaviour guidelines [3, 4]. Physical inactivity will result in nearly 500 million new cases of preventable chronic health conditions worldwide by 2030 if current inactivity levels remain unchanged [5].

In Ireland, public health policy has included PA promotion in primary care through programmes such as the Global Action Plan for Physical Activity, National Exercise Referral Framework, the national structured Chronic Disease Management (CDM) programme and the Making Every Contact Count Framework [6–9]. Introduced in 2020, the national CDM programme provides patients who are diagnosed with certain chronic diseases with biannual appointments that involve a thorough assessment of risk factors and screening for complications as well as structured lifestyle promotion, including PA assessment and advice [8]. Early research reports significant engagement by clinicians with this programme with benefits for patients [10].

Systematic reviews of PA interventions delivered in primary care report small but significant increases for device-measured and self-reported PA [11, 12]. This is reflected in the National Institute for Clinical Excellence (NICE) guidelines that recommend primary healthcare workers give brief advice to increase PA levels [13]. Further, NICE emphasise the need to understand the attitudes of both primary care practitioners and patients to PA promotion in primary care consultations [13]. However, the uptake of PA counselling in general practice consultations is as low as 37.9%, according to a recent systematic review [14]. General practitioners (GPs) recognise the importance of PA promotion and their own role in the process [15, 16], but the reasons for the poor and varied uptake are not clear.

GPs can impact PA levels as they have frequent contacts with patients over time as well as having high levels of patient trust [17]. However, researchers state that the exact role of the primary healthcare professional in PA promotion requires further research [18]. To date, no study has reviewed the literature pertaining to both professional and patient perspectives to understand how patients with different chronic health conditions respond to PA promotion and what would help them become more physically active. Therefore, the research question underpinning this narrative review is what are the experiences of patients and GPs with PA promotion in consultations? Answering this question will provide valuable information on how to optimise PA promotion in general practice by identifying barriers and attitudes as well as strategies to support PA promotion.

## Methods

### Definitions

GPs and practice nurses (PNs) are healthcare professionals working in the primary care setting. Primary care is defined as a range of services designed to keep people well, from promotion of health and screening for disease to assessment, diagnosis, treatment and rehabilitation as well as personal social services [19]. The primary care setting offers a strategic opportunity to implement successful behaviour change interventions [20]. PA refers to any bodily movement involving skeletal muscles and energy expenditure [21]. Exercise is a subset of PA that is “planned, structured, and repetitive and has as a final or an intermediate objective the improvement or maintenance of physical fitness” [21].

### Study design

A narrative review design was developed as this approach offers the flexibility to include studies with different methodologies, also allowing the authors scope to compare and interpret findings from different stakeholder groups [22]. The review research question hinges on stakeholder experience rather than efficacy or effectiveness and the narrative design is suited to this type of research. The narrative review process facilitates in-depth analysis on key papers as opposed to stipulating that all papers that meet the inclusion criteria be included (as per systematic reviews) [23].This “depth over breadth” philosophy was important decision in the development of the review.

### Search strategy

The approach to the search strategy was adapted from the protocol of a previous review designed by one of the researchers (AOR) in conjunction with a librarian [24]. A comprehensive search of the literature was performed through EBSCO Host and PubMed of English-language journals from 2002 to date. This narrative review included both qualitative and cross-sectional studies of healthcare professionals and patient groups in primary care were included. Grey literature was not included (the full list of inclusion and exclusion criteria are outlined in Table 1). While limiting the search to two databases and omitting grey literature may be a limitation of the process, the decision was based on discussion and a preference for a lower volume of studies that were high quality, clinically relevant papers. The search terms used were “physical activity or exercise or fitness or physical exercise”, “general practice or GP or doctor or primary care”, “patients or clients or client or patient or individual or service user”, “perspectives or views or perceptions or attitudes or opinion”, “barriers or obstacles or challenges”, and “facilitators or motivators or enablers”. The search strategy used is outlined in detail in Table 2. The PRISMA protocol was followed, whereby after articles were retrieved and duplicates removed, they were screened sequentially for suitability by title, abstract and then full text [25].

**Table 1:** List of inclusion and exclusion criteria

Inclusion criteria	Exclusion criteria
Original qualitative and quantitative studies involving adult patients’ perspectives on physical activity promotion in general practice	Studies not in the English language
Original qualitative and quantitative studies exploring GPs’ and PNs’ perspectives on physical activity promotion in general practice	Interventional studies and review papers
Cross-sectional studies	Studies not set in general practice

**Table 2:** The search strategy used

Search string 1
“physical activity or exercise or fitness or physical exercise” AND “general practice or GP or doctor or primary care” AND “patients or clients or client or patient or individual or service user “ AND “perspectives or views or perceptions or attitudes or opinion” AND “barriers or obstacles or challenges) AND ( facilitators or motivators or enablers”
Database: EBSCO
Search string 2
(“Exercise”[Mesh] OR “Physical Activity”[tiab]) AND (“General Practice”[Mesh] OR “General Practitioners”[Mesh] OR “Physicians, Primary Care”[Mesh]) AND (“Barriers”[tiab] OR “Facilitators”[tiab] OR “Motivation”[Mesh] OR “Health Knowledge, Attitudes, Practice”[Mesh])
Database: Pubmed
Search string 3
(“physical activity”[tw] OR exercise[tw] OR fitness [tw] OR “physical exercise”[tw] OR “Exercise”[Mesh]) AND (“general practice”[tw] OR GP [tw] OR doctor [tw] OR primary care [tw] OR “General Practice”[Mesh] OR “General Practitioners”[Mesh] OR “Physicians, Primary Care”[Mesh]) AND (patients[tw] OR clients [tw] OR client [tw] OR patient [tw] OR individual [tw] OR service user [tw] OR “Patients”[Mesh] OR “Persons”[Mesh]) AND (perspectives[tw] OR views [tw] OR perceptions [tw] OR attitudes [tw] OR opinion [tw] OR “Health Knowledge, Attitudes, Practice”[Mesh] OR “Attitude of Health Personnel”[Mesh] OR “Attitude to Health”[Mesh] OR “Public Opinion”[Mesh] OR “Attitude”[Mesh]) AND (barriers[tw] OR obstacles [tw] OR challenges[tw] OR “Communication Barriers”[Mesh] OR “Resource-Limited Settings”[Mesh]) AND (facilitators[tw] OR motivators[tw] OR enablers[tw] OR “Motivation”[Mesh])
Database: Pubmed

### Reflexivity

The background of the authors is an important consideration for narrative reviews. Both authors are clinicians (SD, physiotherapist; AOR, general practitioner) and have approached the development of the research question from their personal clinical experience. Determining which papers were included, how findings were interpreted and the significance of those findings were viewed through the clinical lens. Reviewer bias was mitigated by a process of “active acknowledgement” at all stages of the review, including designing and conducting the search strategy and during the several iterations of the paper [26]. An “over and back” drafting style was used whereby earlier sections of the paper were edited as data emerged from the review. This is all in keeping with the narrative review style [22, 23] which encourages the development of opinion from studies reviewed beyond objective presentation and description of data.

## Results

**Fig. 1:**
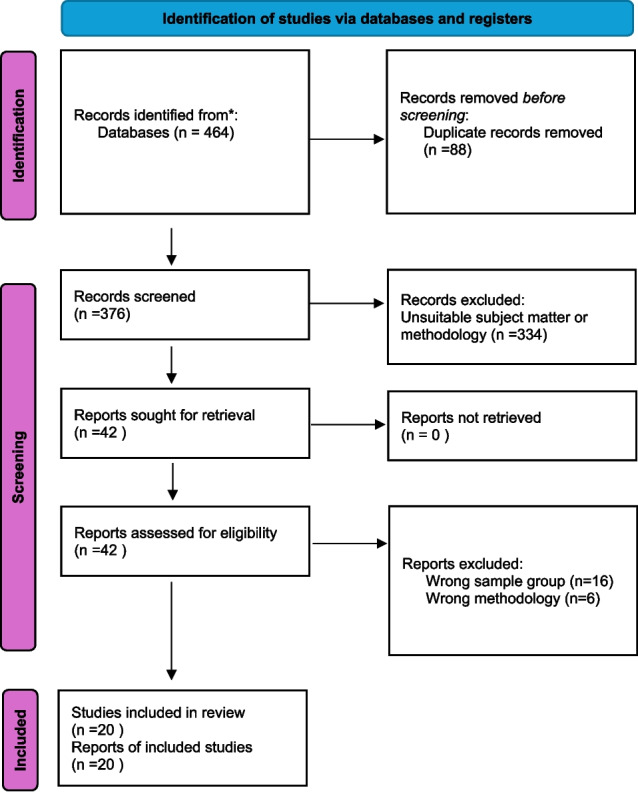
PRISMA protocol

Initial searches yielded 464 papers; the number was reduced to 20 studies after the removal of duplicates and screening out of papers as outlined in Fig. 1.

### Overview of included studies

All 20 studies were set in general practice, including 16 qualitative studies, one mixed method study based on surveys and interviews and three cross-sectional, survey-based studies (Table 3). Eight studies included the perspectives of GPs and PNs only, six studies focussed solely on patients’ perspectives and six studies included perspectives of GPs, PNs and patients. Ten studies had no specific disease focus, and ten studies focussed on specific chronic diseases, including type two diabetes mellitus (*n* = 2), type one diabetes mellitus (*n* = 1), chronic back pain (*n* = 1), COPD (*n* = 1), obesity patients (*n* = 3), MSK injuries (*n* = 1), and cancer (*n* = 1). Sample size of the qualitative studies ranged from five [27] to 74 subjects [28], while study population of the surveys ranged from 117 [29] to 340 [30]. The review included 14 papers from Europe, three from New Zealand, one from Australia, one from South America and one from Asia. The findings were categorised into (1) perspectives of GPs and practice nurses and (2) patient attitudes and experiences.

**Table 3:** Summary of results

1st author, year	Study design	Setting, describe participants, # participants	What were the main findings in the context of our research question 1) To identify perspectives and barriers to PA promotion reported by those working in general practice 2) To explore patient attitudes to and experiences with PA promotion and general practice 3) To synthesise the papers and propose strategies and points of leverage that can support PA promotion
Attalin, V 2012	Cross-sectional study	254 GPs in the South of France Their attitude on PA prescription for obesity was explored	94% reported prescribing PA frequently for obesity Only a minority recommended an amount in line with guidelines Most GPs reported lack of training in PA prescription. Only half wanted to undergo such training Only 1% used online resources and tools but 62% were keen to include it in their practice
Calonge-Pascual, S 2023	Qualitative review of semi-structured interviews	5 GPs and nurses working in Spain	**1) Perspectives and barriers to PA promotion reported by those working in general practice** Health professionals interviewed indicated the need to PA promotion (PAP) and their willingness to participate Nurses showed insecurities around exercise prescription whereas doctors demanded teamwork with other specialists. GPs said they would rather nurses do it but the nurses felt they did not have adequate training Primary barriers reported were lack of PAP knowledge, time, space to assess and institutional support to collaborate with public resources **3) Strategies and points of leverage that can support PA promotion** Both GPs and nurses felt staff training in PAP was essential The GPs group proposed to establish similar strategies as used with smoking or obesity at PHC settings. They stated specifically that they take on the responsibility by getting trained and passing on that training to other staff the same way it was done for smoking cessation
Carstairs, S. A 2020	Explorative study using semi-structured interviews	14 patients and 14 GPs from one UK NHS board Looked at 3 methods (informal signposting, informal active signposting and formal referral) of connecting pts to “jogscotland” a local exercise scheme	**1) Perspectives and barriers to PA promotion reported by those working in general practice** GPs recognised their responsibility in PA prescription but felt they should not be solely responsible and that responsibility lies with the wider community and society to normalise not medicalise PA For some GPs, there were medicolegal concerns about referring to programmes they didn’t know the suitability of For GPs the barriers fell into 5 domains. The first was whether they felt the patient would be receptive to the advice. If they felt the patient would not they often didn’t bring it up. Within this the GPs considered the barriers to exercise for the patient and let this influence whether or not they would discuss PA Lack of knowledge about what opportunities were available was another barrier **2)Patient attitudes to and experiences with PA promotion and general practice** The majority of patients described being open to PA discussions with their GP. For patients GPs raising the topic of PA can help to justify, facilitate, and motivate action to change. Some patients liked the idea of a formal prescription whereas others disliked the feeling of be being “dictated” Patients liked the idea of being connected to PA groups **3) Strategies and points of leverage that can support PA promotion** Both patients and GPs identified the use of formal advertising with things like signs in the practice and leaflet as a passive way to let patients know what is available locally and what it involves. The patients felt being given something was similar to a medication prescription Patients felt a “meet and greet” occasion for potential groups to meet with participants would be a good idea to introduce patients to PA groups
de Boer, J. J 2022	Qualitative, narrative research method storytelling was applied	12 pts and 11 GPs and PNs Adults who participated in a PA-promoting programme focusing on supporting people with one (or more) chronic diseases or at high risk of developing a chronic disease GPs and PNs in primary care	This paper used the COM-B model to discuss its subthemes **1) Perspectives and barriers to PA promotion reported by those working in general practice** Healthcare professionals expressed their inability to help patients with insufficient health literacy Financial, material and logistical factors were mentioned as the prerequisites to good PA uptake according to the GPs and PNs **2)Patient attitudes to and experiences with PA promotion and general practice** Participants reported that trust in the professional and treatment programme is essential to maintain PA. This was achieved when the patient felt understood and trusted the expertise of the GP/PN Patients did not like the discussion of sport and exercise and felt more encouraged when trying just to increase movement and PA Patients noted communication and personal contact as prerequisites to good exercise PA (in contrast to GPS and PNS prerequisites) Patients stated that a temporary interruption of PA, for instance, for medical or social reasons, can be disastrous for sustained PA **3) Strategies and points of leverage that can support PA promotion** Patients reported 5 points that would aid PA promotion 1. Customised PA 2. Increased awareness 3. Meaningful purpose.—linking to patients life and goals can help 4. Group exercise—some patients went to be in a group not too achieve PA but to meet people 5. Success experiences like having positive experience with exercise increased uptake
Dranebois, S 2021	Cross-sectional descriptive study using a questionnaire	152 GPs in French Guiana were asked about their practice in prescribing PA in type 2 diabetic patients 73 GPs responded ages 29–73	**1) Perspectives and barriers to PA promotion reported by those working in general practice** 74% of GPs prescribed PA to patients with T2DM However, only 37% implemented recommendations Majority of GPs felt interested but only 11% were trained Being convinced their recommendations were taken was a huge enabler to GPs for prescribing PA Primary barriers to PA promotion were lack of adapted structure, patients predictable non-compliance, inadequate supports and lack of training and knowledge GPs reported the following as the main reason pts with T2DM don’t exercise: Lack of interest, lack of knowledge on the benefits, few local structures and or remoteness of the structures, physical limitations and co morbidities and finally cost **3) Strategies and points of leverage that can support PA promotion** The main enablers were collaboration between the physician and sports healthcare workers. 84% felt compensation of the cost linked to PA prescription would increase prescription levels
Elley, C. R 2007	Nested qualitative study within mixed methods approach	15 sedentary adults from urban and rural general practices in New Zealand	**2) Patient attitudes to and experiences with PA promotion and general practice** If the advice was delivered discretely, sensitively and was individually tailored, it was more acceptable to patients. Individually tailored approach was important. A lot of participants felt the GPs influence was pivotal as a motivator Primary barriers identified: lack of footpaths, weather, health issues, lack of time and poor attitudes towards exercise were all described as the primary reason uptake was not happening **3) Strategies and points of leverage that can support PA promotion** Patients felt tailored advice with sensitivity, internal motivators, e.g. personal commitment, authority if initiators, e.g. GPs and continued external support were the primary things that helped uptake. They felt a GP or practice nurse prescribing PA gave it a sense of legitimacy
Gillman, T 2018	Mixed method study with interviews and surveys 108 GPs and PNSs completed the survey and 10 were interviewed	117 GPs AND PNs (108 were GPs) completed the survey and 10 were interviewed They were asked about prescribing self-directed exercise for musculoskeletal injuries	**1) Perspectives and barriers to PA promotion reported by those working in general practice** 73.1% recommended self-directed-exercises to patients. 77.8% did this through exercise pamphlets The most commonly used source was arthritis UK General practitioners were positive about the potential for self-directed-exercises to enhance their patients’ well-being They felt participants had clear expectations, e.g. imaging and if exercise was not one of these self-directed exercises would probably not work Time was identified as the biggest barrier. GPs felt if patients had multiple issues exercise was difficult to bring up. In this situation, pamphlets were used **3) Strategies and points of leverage that can support PA promotion** GPs felt better exercise pamphlets would improve the uptake Some mentioned they would be more likely to prescribe exercise if it was clearly listed in treatment pathways
Gustavsson, C 2018	Descriptive study using qualitative content analysis of interview data with both a deductive and inductive approach	18 (10 managers and 8 GPs/PNs) stakeholders in SPAP (Swedish Physical Activity on Prescription)	**1) Perspectives and barriers to PA promotion reported by those working in general practice** All participants perceived that it was important to address health behaviours in patient consultations/encounters, but were not sure that their colleagues felt it was important. (Managers felt GPs didn’t feel they’d a responsibility to do it) The GPs and PNs perceived PA as a health behaviour that was easy to address, as opposed to addressing food/nutrition or alcohol/drugs Most GPs and PNs felt they did not have enough training for PAP The majority of participants did not feel adequately trained in the suggested approach for exercise prescription (SPAP) They also felt it took too much time that they did not have **3) Strategies and points of leverage that can support PA promotion** Need for knowledge and organisational support was identified as the overarching theme with 4 categories, increased knowledge and affirmative attitude among GPs and PNs, clear supportive management, central support structure and local support. The idea of a central coordinator or educator for exercise promotion was mentioned
IJsbrandy, C 2019	Qualitative study based on focus group interviews	12 participants from the Netherlands All participants were receiving or had just received treatment for cancer	**2) Patient attitudes to and experiences with PA promotion and general practice** Participants felt a lot of PA programmes contained fixed elements that were not tailored to their needs or capacities They felt lack of knowledge and skills among GPs and PNs resulted in a lack of qualified information They felt GPs didn’t know what courses to recommend Accessibility was a barrier mentioned with inconvenient times and travel distances etc. effecting participation Lack of information and capacity on courses was identified as well as long waiting lists For many participants, lack of insurance-coverage was a barrier to joining a PA programme, since they did not have the money to pay for the PA programme themselves **3) Strategies and points of leverage that can support PA promotion** They felt group classes with people in similar situations would increase engagement The lack of a sense of responsibility to participate was a main barrier to implementing PA programmes. They felt a referral to an exercise programme would increase uptake
Korkiakangas, E. E 2011	Qualitative study based on video recorded group counselling sessions	74 subjects at high risk of T2DM	**2)Patient attitudes to and experiences with PA promotion and general practice** Pleasure and enjoyment was primary facilitator identified, followed by tools for monitoring one’s own exercise, e.g. technical equipment, next there was encouragement and social support, then health and finally exercise is important value in life, i.e. positive attitude towards exercise Primary barriers to exercise reported were work-related problems and lack of time The seasons and poor weather, health issues and finally no interest in exercise where also identified as barriers
Kosteli, M. C 2017	Qualitative study with thematic analysis of 4 focus groups	26 individuals with a range of COPD severity with an age range of 50–89. 42% had moderate to severe breathlessness, Participants were drawn from the Birmingham COPD cohort study. All were from a primary care disease register	**2)Patient attitudes to and experiences with PA promotion and general practice** This paper looked to highlight intrinsic barriers and facilitators Primary barriers to PA identified The majority of barriers related to the nature of the disease, physical limitations and constrained mobility were key barriers identified. Poor weather was another barrier and participants felt it made them more breathless. Fear of breathlessness, embarrassment and frustration where other barriers identified. Another barrier was participants outlook on exercise, many felt there wasn’t much benefit and had low outcome expectations Primary enablers to PA were identified. Beliefs that exercise is beneficial for COPD patients was a key determinant for engagement in PA. Enjoyment and motivation where another two key enablers. This studied highlighted that some participants felt taking care of a family member as a reason to be physically activate was a motivator with lower psychological well-being compared to autonomous motivation. Having a personal routine and unique way of pacing was a novel enabler highlighted in this study. The feeling of accomplishment was highlighted. Finally the social aspect was a key factor highlighting the importance of emotional support
Larsson, K 2023	Qualitative design with focus group discussions	Based in Swedish primary care. Looked at perceptions of GPs and PNs who work with metabolic risk factors with regard to increasing PA levels 9 physiotherapists, 10 physicians and 5 nurses participated in 6 digital focus group discussions	In short highlights the complexity of PA promotions with personal and organisational factors at play **1) Perspectives and barriers to PA promotion reported by those working in general practice** They found discussing PA much easier than discussing other lifestyle changes. Participants felt it was fun and rewarding to be a part of positive changes in patients. They said they struggled to engage in PA prescription in patients who weren’t receptive. All three professions felt the physicians advice was taken the most serious by patients GPs and PNs felt that patients knowledge, self-awareness and a desire to increase PA where indicators as to whether PA prescription would be successful. Another barrier in this sense was the patients feeling of guilt, they felt it led to helplessness and low self-efficacy Lack of time and resources to increase PA was the primary barrier mentioned. Another factor was the economic compensation, preventive work is not recognised and therefore clinics would not receive compensation **3) Strategies and points of leverage that can support PA promotion** They reported personalising the PA prescription as one of the most important factors in increasing PA They felt tools played. A positive role and things like step counters hand outs and group classes could be used They felt having more avenues to refer to such as sports groups or clubs would increase the prescription and uptake of PA
Lascar, N 2014	Qualitative study based on semi-structured interviews	26 adults with T1DM 12 Fs 14 MS Ages 21–65 Questions looked at their PA and exercise behaviours as well as the barriers and facilitators	**2) Patient attitudes to and experiences with PA promotion and general practice** Advice from a health or fitness professional: This was the most possible intervention. Having tailored input was a key facilitator Motivational support: This was the least popular of the options. People felt it may feel childish or nagging 4 main barriers were identified 1. Health and medical Diabetes-related issues were highlighted—fear of hypoglycaemia was mentioned but wasn’t a clear cited reason to not exercise Some non-T1DM issues such as arthritis were listed as barriers 2. Time work and lifestyle Lifestyle factors were a far greater barrier identified than their diabetes. Half of participants noted lack of time as a barrier. A lot noted that it wasn’t a lack of time but rather poor planning and prioritisation. They felt that when combined with things like poor weather lack of time was used as an excuse not to exercise 3. Social and personal. Lack of motivation and being “lazy” were listed by around a quarter of participants. Embarrassment was mentioned by 3 participants 4. Environmental Access to facilities was identified by some participants with cost being an issue. Weather was also mentioned as an issue **3) Strategies and points of leverage that can support PA promotion** Free passes or incentives: although only a minority mentioned cost as a barrier a lot felt free access to facilities would be a big incentive Training partners was mentioned people felt having someone to train with was a facilitator Some felt health benefits were a facilitator Enjoyment was another important facilitator Half the participants supported the idea of attending group exercise classes with the social aspect being the primary benefit. The main points against it depended on the nature of the group and how participants would be perceived. Likeminded groups were the primary suggestion in this category
Leemrijse, C. J 2015	Written questionnaire	340 Dutch GPs responded 4 main aims of the questionnaire 1) perceived role of the GP in promoting PA 2) Whether GPs referred to healthcare professionals or sports clubs 3) Barriers and motivators to referral outside the healthcare setting 4) Involvement of GPs in structural collaboration with local clubs to promote PA	**1) Perspectives and barriers to PA promotion reported by those working in general practice** Half of the GPs thought that they had an important role in stimulating PA, while the other half considered their role present but “limited” GPs referred to a local exercise facility because they thought that PA should take place outside the healthcare setting When referred most patients were sent to a physical therapists but local exercise facilities were also mentioned Limited motivation of the patient was the primary barrier to advising patients to exercise as well as reduced health status Limited financial possibilities of patients was another mentioned barrier **3) Strategies and points of leverage that can support PA promotion** Positive experiences of patients, affordable offers and information on local facilities were seen as the main promoting factors for referral to exercise professionals
Lindner, N 2023	Qualitative study based on semi-structured interviews	Interviews were split into 2 groups. 12 GPs and 14 patients with CBP (based In Germany) Patients must have had at least 3 contacts with the GP in 6 months about the CBP	**1) Perspectives and barriers to PA promotion reported by those working in general practice** GPs stated that they did not have enough time in their daily work routine for giving advice to patients on PA Some of the GPs said that they did not feel confident advising patients on PA Some GPs expressed their frustration in the treatment of patients with CBP. They often believe patients do not follow their PA recommendation. GPs identified peoples bad habits and lack of drive as a barrier to PA. Insufficient ability to maintain a routine of PA was identified as a barrier Co morbidities were listed as a limiting factor by GPs In terms of external factors GPs Insufficient regional availability was identified. COVID restrictions were listed by both groups as a factor that was effecting access to classes **2) Patient attitudes to and experiences with PA promotion and general practice** Overall patients had a positive opinion on PAP and the positive effect it had on their pain Some patients expressed feeling stigmatised and frustrated Insufficient ability to maintain a routine of PA was identified as a barrier by patients as well. Patients reported feeling stigmatised, frustrated and lacking support. They also felt anxiety around exercise created avoidance behaviours “discrepancy in perceptions of the doctor–patient relationship and resulting negative emotions is highly important.” Patients listed comorbid conditions as a limiting factor to PA **3) Strategies and points of leverage that can support PA promotion** Enjoyment was highlighted by both groups as a key factor GPs mentioned how PA should be suggested as easy to carry out, e.g. going for a walk Group exercise classes was suggested by both groups. A summary of available classes was seen as a good idea
Martínez-Ramos, E 2015	Descriptive-interpretive qualitative study	Based in Spain. 23 patients with overweight or moderate obesity who sit for at least 6 h a day. 10 in-depth semi-structured interviews were completed	**2) Patient attitudes to and experiences with PA promotion and general practice** Barriers to reducing sedentary time included work and family routines, lack of time and willpower, age and sociocultural limitations. Facilitators identified were sociocultural change, free time and active work, and family surroundings Participants recognised the abilities of GPs and PNs to provide help and advice, and reported a preference for patient-centred or group interventions They did not feel one intervention was enough to break the habit Participants did not recognise the consequences of prolonged sedentary behaviour **3) Strategies and points of leverage that can support PA promotion** Participants felt It would be advisable to make the public more aware, informing them about the ill effects of being seated for many hours Participants felt follow-up after the intervention was important be it an email, phone call or appointment with the practice nurse
Patel, A 2011	Qualitative study based on interviews	15 GPs in New Zealand who issued Green prescriptions The interviews looked to explore the GPs perceptions of the Green Prescription programme “A Green Prescription is written advice from a health professional (usually your doctor or practice nurse) to be active and improve your diet. It is a support service that helps you to improve your health and feel better at the same time.”	**1) Perspectives and barriers to PA promotion reported by those working in general practice** GPs felt exercise prescription was very useful in managing patients weight and health. They often referred patients with multiple risk factors for certain modifiable diseases. They also saw the benefits of such referrals for patients suffering with depression. Primary reason was weight management and pre-existing conditions Time was the only barrier to prescription for GPs. Patient often present with multiple issues which left no time to discuss PA. Many GPs said they passed on this role to the nurse to save time **3) Strategies and points of leverage that can support PA promotion** GPs’ perceived benefits of the Green Prescription programme specifically Two main associated subthemes emerged: (i) a non-medication approach to a healthier lifestyle, and (ii) the support benefits of PA i) A lot of GPs liked the Green Prescription allowed patients to make healthful changes that didn’t involve medication ii) Patients were getting referred to a support councillor, GPs felt these councillors had the skills and time to fully support patients in initiating increased levels of PA. They also stressed that having a specialised support increased patient safety as they could be monitored
Patel, A 2012	Qualitative study based on interviews	15 GPs in New Zealand who issued Green prescriptions The interviews looked to explore what GPs felt where the primary barriers to exercise for patients over 65	**1) Perspectives and barriers to PA promotion reported by those working in general practice** GPs said patients often reported the very reason they need to exercise as why they don’t exercise, e.g. SOB, OA or high BP. Other limiting conditions were things like skin rashes after swimming Transport constraint was one of the most identified barriers with GPs reporting patients couldn’t get to the venues of organised classes GPs identified chronic health conditions, fear of injury, transportation constraints, set routines and lack of confidence as being barriers that some of their older-aged patients have encountered when considering whether to become more physically active **3) Strategies and points of leverage that can support PA promotion** One thing GPs mentioned to try increase uptake was repetition, they felt bringing it up at regular consultation often resulted in patients becoming more comfortable with the idea
Wattanapisit, A 2019	Qualitative study	17 GPs based in Thailand working in district hospitals. Saw outpatients only	**1) Perspectives and barriers to PA promotion reported by those working in general practice** Counselling practices: GPs concentrated on high risk patients with non-communicable diseases such as hypertension and obesity when deciding who to prescribe PA too. The second most common was musculoskeletal injuries such as osteoarthritis GPs used the FITT principle when discussing PA with patients but often intensity was overlooked Primary reason for PA counselling: To improve patients’ quality of life—improves health and is free The best non-pharmacological tx for many NCDs Barriers to PA counselling: Time constraints was one of the largest barriers Lack of knowledge and skill. GPs mentioned finding it hard to prescribe when someone has a disease, poor communication skills was also mentioned as a barrier Discontinuity of care: GPs felt if they don’t have the same patients it’s hard to encourage pa **3) Strategies and points of leverage that can support PA promotion** Training in PA counselling for GPs The idea of premade prescription sheets was mentioned, low literacy levels in Thailand was identified as a barrier to these Training other staff such as nurses or physios was also mentioned
Winzenberg, T 2009	Qualitative study based on semi-structured interviews	15 GPs selected from South Tasmania to include a range of demographics	**1) Perspectives and barriers to PA promotion reported by those working in general practice** GPs recognised the importance of assessing PA levels but instead of asking everyone they screened those they felt most at risk with conditions that would benefit from increased PA Several GPs placed a higher priority on assessing smoking behaviour than PA Depth of assessment and definition of sufficient PA levels varied among GPs Primary barriers were the time needed for an adequate assessment and lack of time to deal with inactivity once its identified

### Experiences of GPs and PNs

#### Role in PA promotion

GPs and PNs perceive PA as an important health behaviour to promote among all patients. GP and PN study participants of the studies under review stated that PA is an important part of the treatment of musculoskeletal conditions [29], T2DM [31], obesity [32], metabolic risk factors [33] and general health [34]. Most GPs and PNs believe that they play an important role in this PA promotion [27, 30, 31, 35] and that discussing PA is easier than discussing other lifestyle changes such as healthy eating or unhealthy habits such as alcohol and drugs [33, 36]. GPs and PNs found it enjoyable and rewarding to help patients make positive behavioural change relating to PA [33]. A strong theme common to most papers was that PA promotion should be shared among professionals, including “exercise specialists” [27] as well as non-professionals in the community and wider society to normalise and demedicalise PA [35]. GPs and PNs are confident bringing up the subject of PA and supporting the patient to make a change but beyond this, other resources must be accessible to which the medical professionals can signpost, thereby demedicalising the process.

#### GP and PN barriers

1. Lack of time
  Lack of time was the most frequently mentioned barrier by GPs and PNs [27, 33, 34, 36–39]. It is challenging to introduce the subject of PA when the patient presents with multiple issues. Time constraints can be overcome by sharing the responsibility of PA promotion with others [27, 40].
2. Systemic and organisational barriers
  Lack of institutional support [27], not receiving financial compensation [33], and medicolegal concerns about referring to programmes with uncertain suitability for patients [35] were identified. Two papers cited being remote from structures and resources as logistical barriers [31, 37]. Financial material and logistical factors falling into place were prerequisites for good PA uptake highlighted by GPs and PNs [41].
3. Lack of knowledge and training
  Some GPs and PNs did not feel confident in PA promotion [37]. Specifically, they reported lacking knowledge and skill on PA promotion [31, 38] and available opportunities for exercise prescription [35], with some expressing a need for more training to address this [27, 38].
4. Patient-related factors
  GPs and PNs reported that their interventions were limited by poor motivation, self-awareness and desire among patients [30, 31, 33, 37], leading to frustration from GPs and PNs [37]. When non-compliance with PA advice became a recognisable pattern it disincentivised GPs and PNs’ PA promotion [31]. Traits among patients were identified by GPs and PNs that made engaging with PA promotion challenging, including patients who weren’t receptive [33], communication deficits [38, 41], feeling of guilt leading to helplessness and low self-efficacy [33].

#### Patients’ experiences

1. Receptiveness to PA advice
  All but one paper on patients’ perspectives reported a positive disposition to PA promotion [17, 35, 37, 42, 43]. Patients are receptive to GPs or PNs raising the topic of PA [35], provided the advice is delivered sensitively and is individually tailored [42]. Individually tailored advice was favoured in contrast to motivational support which patients said felt like “nagging” and made them feel “childish” [17]. Some participants recounted feeling stigmatised, frustrated and lacking support [37]. The benefits of a personalised, supportive and sensitive approach were described well by one patient in Elley et al:
  The fact that it was a personal approach, and it was so nonjudgmental... There’s no way I’m going to be treated like an idiot... and their sensible handling of what was obviously a sensitive issue for me was really the thing that got me and held me. [42]
2. Patient-reported barriers to PA
  Lack of time was the primary barrier to PA mentioned [17, 28, 42, 43]. Patients in Lascar and colleagues reported lifestyle factors such as lack of time as a far greater barrier than their diabetes but some patients in the same study believed that poor planning and prioritisation were the primary factors and that time constraint simply an excuse [17]. Poor self-efficacy emerged as a barrier, among patients who perceived themselves as “lazy”, or that they lacked motivation or routine [17, 44]. Further insight was observed in:
  So why do we need to do all this exercising at our age now, I don’t really think you can get a massive amount of benefit at our age. Younger people yes, but our age no [44]
  I am not interested in exercising anymore.... [28]
  On the other hand, participants in the study by Kosteli and colleagues, having a personal routine was an enabler to sustained PA [42, 44]. Poor weather was mentioned in four papers [17, 28, 42, 44].
3. Patient preferences for PA promotion
  Patients prefer a personalised, patient-centred approach [43]. Multiple studies highlighted meaningful personal goals as a tool to motivate patient to exercise [41], such as reaching a certain number of laps in the swimming pool or being fit enough to attend a loved one’s birthday party. Participants in the study by De Boer and colleagues stated that trust in the GP or PN was essential to maintain PA levels, and that trust was achieved when patient felt understood by their GP or PN [41, 44]. Patients had a desire for community, with group classes being one of the main preferences raised [17, 35, 41, 43]. Social support and enjoyment help patients maintain PA levels with many patients describing the social aspect as the primary benefit [17, 28, 44]:
  I’ll play any sport just for the social side of it; yeah I do enjoy that, that’s a motivation. [44]

#### Interpretation of stakeholder experiences

**Fig. 2:**
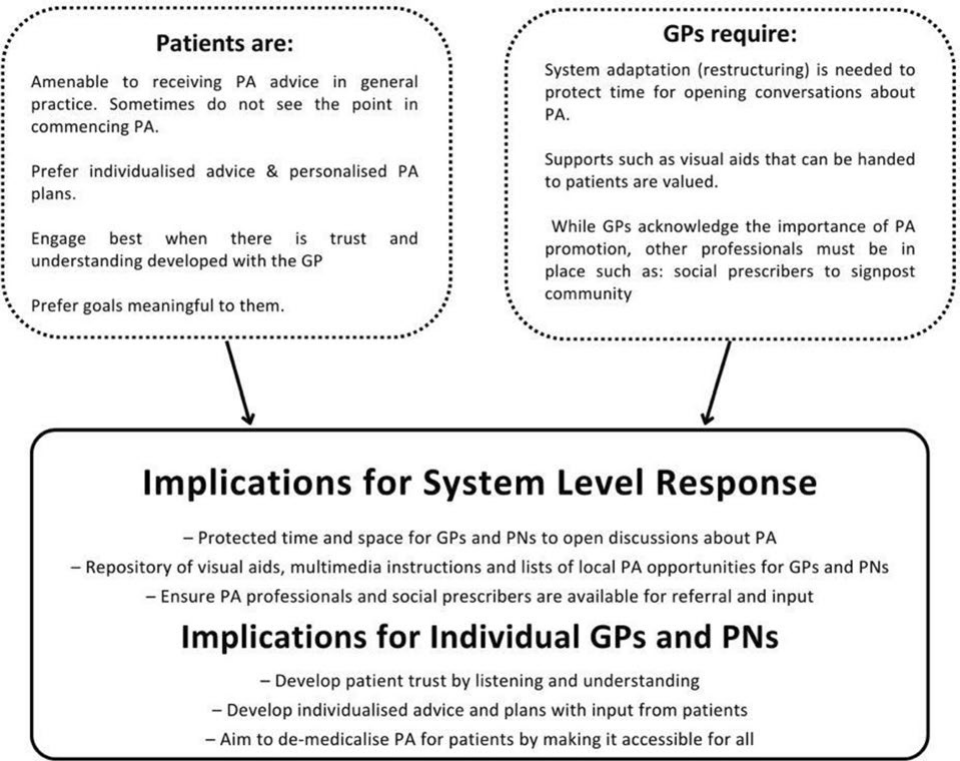
GP’s and patient’s perspectives

Consistent across papers and regardless of stakeholder group was the finding that PA promotion is important and should take place in general practice consultations (Fig. 2). However, the type of approach taken is the key deciding factor on how the patient responds. The chief role of the GP or PN is to identify the physically inactive patient, understand the barriers specific to that individual and to collaborate with the patient to help them find ways to engage and maintain PA behaviours. This “demedicalised” approach to PA promotion is acceptable to patients and is acknowledged by GPs and PNs as being part of their duty of care. Some may require further training to do this but more important points of leverage identified include addressing systemic barriers (especially by protecting time), involving community resources such as exercise professionals to whom the GP/PN can signpost and developing a suite of resources for use within the consultation, e.g. exercise prescription templates. These are discussed further below.

#### Strategies and points of leverage to support PA promotion

1. Addressing systemic barriers
  Ultimately, optimisation of PA promotion in general practice requires support at a health systems level. If GPs are under time pressure dealing with huge acute patient lists, PA promotion is unlikely to become embedded. To address this, systems for ensuring that GPs have protected time and space to raise the topic of PA and to delve into patients’ perspectives and barriers. In one study, 84% of GPs and PNs believed compensation of the cost linked to PA prescription would increase prescription levels [31]. There was desire for an evidence-based central resource with self-directed exercises and guidelines on how to prescribe them. GPs stated they’d be more likely to prescribe PA if it was clearly listed in treatment pathways:
  if there is evidence we want to be following it. [29]
  Gustavson and colleagues expressed the idea of a central coordinator or educator for PA promotion [36]. Patients in Martinez and colleagues’ study stated that more public messaging campaigns are vital to raise awareness of the dangers of physical inactivity [43].
2. Collaboration with community resources
  Group PA was one of the primary preferences for increasing PA levels mentioned by patients. This is echoed in their suggestions for things like classes with people in similar situations [17, 45], financial incentives to attend classes [17, 30] and more awareness of classes available in the community [35, 37, 41]. GPs and PNs believe that professional PA counsellors in the community have the skills and time to fully support patients to increase PA [40]. Collaboration was identified as the greatest facilitator to PA prescription by Dranebois and colleagues [31]. Similarly, GPs identified the following to support their role in PA promotion: information on where to signpost patients, collaboration with exercise professionals and materials that could be given as a resource to patients [27, 30, 35].
3. Use of structured PA prescription tools
  Patients express a clear desire for formal exercise prescriptions and signposting to exercise classes [45]. Patients felt being given “something” was similar to a medication prescription [35], providing a sense of legitimacy [42]. Exercise prescription sheets were suggested by Wattanapisit and colleagues but low literacy levels (in Thailand) were identified as a barrier [38]. This highlights the need to adopt different approaches suited to the socio-economic status of the patient groups being targeted. While some GP participants were interested in exercise prescription [29], this is not a finding common to most of the studies and it is noteworthy that self-selection of the most enthusiastic PA promoters is a potential source of bias among many of the studies. The strongest opinion common to GP participants across studies is a call for exercise specialists or social prescribers to whom GPs can refer after they have started the discussion on PA.
4. Improved training and education for GPs and PNs
  Training in exercise prescription was suggested to improve PA promotion by some GPs and PNs [36, 38]. GPs interviewed by Calogne and colleagues suggested it would work best if they received training and passed on training to other healthcare providers and PNs similarly to what was done with smoking cessation [27]. However, in another study half of the participants in Attalin and colleagues did not want to undergo training in PA promotion [32]. This likely reflects the immense time pressures reported internationally on GPs and practice staff.

### Critique of studies

Sampling bias was cited as a weakness in multiple studies as participants with a predisposition for PA were selected [28, 30, 35–37]. Gustavasson and colleagues interviewed stakeholders of SPAP (Swedish Physical Activity Program) including coordinators of the scheme [36] and similarly, patients in Koriakangas’ study had already signed up to an exercise promotion class [28]. Self-selection of study participants may result in an overestimation of the positive attitudes of GPs towards PA promotion [30]. Larson and colleagues lacked an equal gender distribution failing to recruit enough males; Martinez and colleagues also included predominantly females [46, 47]. Lascar and colleagues recruited from a single practice, yielding a cohort who were all under the care of clinicians with a similar positive bias to PA; it also resulted in lack of ethnic diversity as highlighted by the research team [17]. Some studies involved GP participants who had access to unique exercise prescription programmes, thereby limiting their transferability to other settings [34, 36], whereas GP participants elsewhere reported an average consultation time of 3.8 min [38], a factor which understandably would heighten the effects lack of time has on PA promotion compared to the Irish average of 13.7 min [48].

## Discussion

### Synopsis of review findings related to the research question

The central research question of this review is what are the experiences of patients and GPs with PA promotion in consultations? Studies involving patients generally reported a positive disposition to PA promotion in the consultation provided it was conducted in a sensitive manner, tailored to the experience of the individual patient. Meanwhile, GPs and PNs were receptive to PA promotion but identified several barriers to successful implementation. The central answer to the research question was the need for GPs/PNs to adopt a collaborative approach with their patient to demedicalise PA and to find ways to make it more accessible for them.

### Comparison of findings with evidence and theory

The finding that some GPs and PNs find it disheartening when time is invested in PA promotion among patients who are not receptive can be better understood through the lens of the transtheoretical model of behavioural change [49]. This model identifies a circle of mindset stages that must be understood by the clinician. A patient in the “precontemplation stage” is not ready to make a change and is unlikely to respond to advice so brief information may be more appropriate. Patients with low self-efficacy were identified by this review as a challenging group for PA promotion. Bandura’s social cognitive theory of PA places self-efficacy at the core of behavioural change; techniques such as goal-setting, identifying and planning for barriers can help patients to improve their self-efficacy [50]. Many of the participants in the studies reviewed here had at least one chronic disease and some perceived themselves to be unfit for or to have nothing to gain from participation in PA. Negative self-image and lack of understanding on the benefits of PA are recognised barriers to PA participation [51]. High-level evidence clearly demonstrates that the greatest health gains from PA are seen among completely physically inactive individuals becoming slightly more active, i.e. going from doing nothing to something (even if they are still well below the recommended daily PA guideline recommendations) [52]. This is an important consideration for general practice where those with multi-morbidity and the least physically active attend the most [53].

### Strengths and limitations

This paper is not intended to be a systematic review and some papers may have been missed including grey literature. The authors’ priority was to ensure the highest quality papers were included so only peer-reviewed, published papers in scientific journals were reviewed. The authors acknowledge other insights may have been missed. The search was completed using PubMed and EBSCO databases so relevant papers may have been missed. Another limitation was that only studies in English were included. As previously stated, sampling bias, restriction of recruitment to single centres and under representativeness of certain socio-demographic characteristics are common to many of the papers and thereby limit the generalisability of the review findings.

### Implications of this study

The findings of this review have important implications for clinical practice, professional training, policy and research.

### Implications for clinical practice

General practice consultations, involving GPs and PNs, have the potential to act as a vehicle for PA promotion. This study demonstrates that both patient and professional groups recognise the relevance of PA promotion for this setting. GPs, through repeated contacts with their patients, have their trust and have a unique insight and understanding of the patient to approach PA promotion so their patients can make timely and sustainable behavioural change. There is a need and great scope for widespread uptake of PA promotion in this setting. Policy and training must adapt to reflect this need.

### Implications for professional training

Several papers in this review involving GP/PN participants recommended training in PA promotion, while some papers conducted with patients suggested a more sensitive, individualised approach. Continuous professional development (CPD) is an obvious means to deliver this. The authors suggest that practical, case-based vignettes incorporating the principles of behavioural change applied to typical cases would be of most use and interest to GPs/PNs. Useful resources are already available and could be disseminated through CPD groups.

### Implications for policy

Research on this topic conducted almost 30 years ago identified organisational barriers, particularly time, as key challenges to PA promotion in general practice. Positive developments in recent years, such as the national CDM programme in general practice in Ireland, have changed the structure of general practice, facilitating time for PA promotion (among other health behaviours). However, this pertains only to people who have been diagnosed with one of six specified chronic conditions. A clear and unanimous message from this review is that PA promotion cannot be optimised unless protected time is available. Further restructuring of general practice is needed to facilitate PA promotion of those identified as being at risk of developing chronic disease as well as patients with other chronic conditions, such as hypertension, and mental health conditions such as depression.

Any policy changes involving general practice should be co-ordinated with other departmental and societal policies to ensure an all of system approach, as recommended by the WHO “all systems” strategy [54]. The findings of this review are in keeping with international best practice of a cohesive intersectoral strategy for PA promotion, whereby the responsibility for PA education and awareness is shared with public health, the corporate sector and education. Furthermore, this paper indicates that effective PA promotion in general practice will require the availability and access to exercise professionals in the community. Sharing knowledge of these resources and ensuring they can be accessed by patients without bureaucratic obstacles should be a priority for planning.

### Implications for research

While this review reports on the appropriateness of PA promotion in general practice and suggests ways to optimise it, future research is required to understand which approaches are most effective, if different approaches should be taken for people with different chronic conditions and how best to measure behaviour change over time. While the benefits of PA for people with chronic conditions are reported [2], very little evidence generated from general practice on specific chronic disease groups is available.

Future research is needed exploring the effects of PA promotion in general practice on long-term (beyond 3 years) patient outcomes such as pain, quality of life, hospitalisations and daily functioning. The aforementioned programme has great potential as a setting for any such trial as the continuity of care general practice provides makes it a natural setting for long-term follow-up of PA benefits experienced by patients. The roles of different professionals such as physiotherapists, nurses and exercise professionals in PA promotion need to be further investigated to establish how they can best support GPs in the effort to improve PA uptake by patients. Future research must encompass all of society and studies included in this review and an earlier review of PA trials in general practice report inadequate representation from lower socio-economic groups and ethnic minorities [17, 24].

## Conclusion

PA promotion in general practice is acceptable to patients, PNs and GPs. Patients report more favourable experiences with individually tailored approaches to PA promotion. Protected consultation time is needed to understand patients’ attitudes and to provide a demedicalised approach whereby the clinician works with patients to make PA participation more accessible for them. Practice-based, educational and policy initiatives have been identified to optimise the environment for effective PA promotion.
